# Central Dental Association of Northern New Jersey

**Published:** 1893-07

**Authors:** 


### CENTRAL DENTAL ASSOCIATION OF NORTHERN NEW JERSEY.

### DISCUSSION ON DR. G. LENOX CURTIS'S PAPER.

(For Dr. Curtis's paper, see page 486.)

*L. Ashley Faught*, Philadelphia.—I wish to compliment our good brother who has so ably presented this subject to us this evening. It is a great pleasure to listen to a paper so well written; and though I may differ with him in the main conclusion, I most heartily agree with him in all that he has said in advocacy of anything leading to higher professional attainments. Post-graduate study is a subject in which I have been interested for many years, as long as—perhaps longer than—any one present here to-night, for I see that the idea was sprung upon the profession in its literature at the organization of the Post-Graduate Dental Association of the United States, in the year 1889. The only reference I can find before this is an article written by myself in 1885, and published concomitantly with one by Professor Barrett, in the same year. This article of mine was the culmination of a personal attempt in 1880 to start such a school as a private enterprise. Thus considering the subject for a period of over twelve years, I entertain views to-day diametrically opposed to those of my first conception. I will in a few words try and tell you the reason for such change. In the first place, we should note that post-graduate study involves three phases,—a mental development, a manual development, and that happy combination of the two, a higher professional development. I will consider each in turn. When I was a young graduate of dentistry, filled with the fire and vigor of youth, and animated by the laudable desire to succeed, I wrote upon my tablets that success was to be obtained only by "reading like smoke." The midnight oil was burned, and early candle-light was used, even the best part of sunlight was devoted to study, seeing that the slow growth of practice left many of its brightest hours unoccupied. As a natural result, I was not only up at all hours, but I felt a manly pride in being "up" upon all subjects. The college habit of acquisition was never lost, but the mystery to me was why men who had been twenty years or so in practice did not seem to be able to keep pace with me. The mystery now has been solved; experience in this matter is the best teacher. I now know—what they then knew—that when one is in full practice, busy each day with its full duties and cares and worries, the physical strength and the mental strength become so sapped that it is well-nigh impossible to do more than that study necessary to the proper conduct of the cases committed to our care.

The appropriate deduction from this is that the value of post-graduate study in the development of a theoretical knowledge is at a minimum at the time of professional career where it is the most needed.

A word now regarding "manual development." Upon this point the essayist of the evening has laid special emphasis. I think it is admitted as an axiom, in all the arts and sciences involving digital execution, that unless one possesses physical endowment, training the best and cultivation the most constant will never develop power beyond a certain point. It is true that much of the awkward and crude may by special drill be removed, but the finer touch so necessary to a finished service, as in crown- and bridge-work, continuous gum-work, or the construction of regulating appliances, etc., can never be attained where native qualities are deficient. The few and not the majority possess these native qualities. Post-graduate instruction, therefore, in this direction is of limited possibilities.

The higher professional development remains now for our consideration. This undoubtedly is the most worthy aim of post-graduate study. Here, in the main, will lie its value,—making better that which is poor, and perfecting the few destined to be shining lights; but to my mind, gentlemen, post-graduate study can never repair the loss the profession has suffered by the abolishment of preceptorships. The absence nowadays of preceptorial teaching I believe to be a positive loss to a dental career, and one which can never be replaced. The foundations are everything, and cannot be too carefully laid. Time spent here is well spent in grounding principles. No spring and fall courses at colleges can take its place. Statements to the contrary in college announcements are a blandishment and a deception. It is ante-college training and not post-graduate study that is the demand of the hour. Colleges should be provided with aged and experienced demonstrators instead of young and recent graduates in this important department. The attention here advocated cannot but result in higher professional development. Refinement of material before and during instruction will produce a depth and richness in the finished product that no gloss and varnish applied afterwards to the crude can ever even approximate.

*Dr. George E. Evans.*—Of the many subjects of an educational character presented for the consideration of the dental profession, none at present is more deserving than post-graduate study. As to what should be the standard course and length of study to properly educate and entitle the dental student to receive his degree are questions which, after years of discussion, have finally been decided, and the decisions have been accepted by all recognized colleges.

The benefit of these innovations is already being felt, and the future results will be to restrict the practice and generally elevate the grade of dentistry by classification in a profession. Impartially considered, the educational advantages that the average student has availed himself of by the time he has graduated consist actually of only a general, not a thorough, comprehension of the subject of dentistry. The future of the young practitioner is thus left to himself, as his alma mater has sent him out in the world as his own master. Then, or a little later, if he is progressive, the subject of post-graduate education will naturally engage his attention. The value of post-graduate instruction, in my opinion, has been presented by the essayist of the evening in a very liberal and concise manner. Facts and collateral matter set forth show a most careful consideration of the subject; therefore, my discussion will partake more of the form of endorsement and comment than criticism.

That until within the last few years many students were graduated from dental colleges and entitled to practise the science and art of dentistry only partially educated, is undeniable, but at the present time this, I consider, belongs to history. An elder profession, like the medical, when presuming to discuss this subject in relation to dentistry, should not forget that it has much to consider respecting its own colleges, as to the manner favored students in some instances have been coached in their studies and favored in their examinations on subjects involving a knowledge of surgery and methods of procedure in practice, which leaves it open to comment and criticism on the subject of "partial culture." But, owing to the educational standard adopted by the National Association of Dental College Faculties, the stringent regulations enforced, and the extension of the term of study from two to three years, and also to the watchful eyes cast and examinations required by State Boards of Dental Examiners, the day when dental students can graduate easily, or receive license to practise unqualifiedly, is past. That a student in a three-years' course at a dental college will receive a fair education in the scientific branches will be acknowledged, so must the fact that a large proportion of his time will be spent in their study. But in dental art and mechanism his education will be more theoretical than practical. In short, he has been taught how he should practise dentistry, but he has yet to learn by experience how it is to be done successfully. This experience will demonstrate to him how much he knows and what skill he has to acquire before he can assume the rank of an operator or expert in any one of the special branches.

The object principally of extending the course of study to three years has been to give, during the third year, better manual training and practical education to students before graduation. But even this extension of time, especially when the curriculum of study has been somewhat extended, and a practical knowledge of such a branch as crown- and bridge-work is required, as it is in the leading dental colleges of this country to-day, leaves but limited time for the acquirement of skill.

Under these circumstances, after graduation and a brief term of practice, it is that the subject of a limited course of post-graduate study and the benefits that could be derived from it will become apparent. To be a successful practitioner the dentist as an operator must be master of what he undertakes, so as to inspire confidence in his patient, insuring for him the best results, in their broadest sense.

Most all practitioners of dentistry favor one special branch more than another. This preference usually takes time to demonstrate itself. A limited course of instruction of a high grade in some preferred branch, in such cases, must necessarily be fraught with interest, and prove advantageous.

The essayist refers to those who entered on the practice of dentistry without a college training who would be benefited by a post-graduate school or a practitioner's course. There also might be included those whose attendance at college was only of a nominal character, sufficient to secure a degree, and a few who graduated many years ago, to whom more modern methods of procedure in practice could be demonstrated advantageously.

The idea of making post-graduate instruction obligatory, I think, would not be proper. The certificate of graduation is one entitling its possessor to practise. Graduates become scattered, in some cases even to foreign countries, and the actual need of post-graduate study in after-years will largely depend on the natural ability of the dentist, the attention he gives to matters which will tend to advance him in knowledge, the character of the field of his practice, and the professional atmosphere in which he moves.

That we have not a post-graduate school as yet, constituted with a corps of instructors such as the essayist describes, is admitted. There are various reasons for this. The services of those learned instructors are not to be easily influenced, bought, or commanded as their time is already occupied to great pecuniary advantage. The preparation and delivery of lectures and the giving of clinics would require considerable time that could not be given for little or no remuneration.

One reason why post-graduate schools have not been added more generally to colleges is that every liberal college is more or less influenced by the wishes of its alumni. The alumni of dental colleges are, as a rule, not favorable to post-graduate schools, as ordinarily conducted, as their advantages are open to all classes of practitioners. In short, the college graduate does not care to return to study in the same ranks with practitioners who are non-graduates, or of questionable standing. In these respects, and in others which might be mentioned, the privileges of a post-graduate school should be liberally but properly restricted, so that its object could not be perverted.

The essayist, in my opinion, very correctly infers that but a small number of practitioners are well versed in the various branches of prosthetic dentistry, especially in crown- and bridge-work. Respecting the invidious reflection that many endeavor to cast on prosthetic dentistry, I will state that when I hear a dentist in a boastful sort of manner remark, "I do no mechanical work," I frequently think, Is he naturally endowed with or possessed of mechanical skill required for the construction of an artistic piece of work, or does his whole embodiment of soul dwell in just that *one* thing in dentistry, the operation of filling a cavity in a tooth? For who will say, who will *presume* to say, in this day, at this period of an advanced state of dental art, that he who can fill a tooth, an operation the difficulties of which are reduced to a minimum in comparison to what they were thirty odd years ago, by improvements in instruments and the use of rubber dam instead of napkins, is to be classed as working in a sphere where more talent, knowledge, and skill are required than in gold-plate and continuous-gum work, the difficult operations presented in crown- and bridge work, and oral deformities, which involve collaterally much that is included or connected with operative dentistry? In presenting the subject of post-graduate instruction, Dr. Curtis has omitted reference to the Post-Graduate Dental Association of the United States, which, as set forth by its circular, is an organization solely engaged in the work of educating dentists. By different courses of reading it aims to reach every class of dental practitioner. These courses of reading are divided into pre-graduate and post-graduate courses; the one designed for the non-graduate, the other for the graduate. The classes organized and to be organized are as follows: A, B, C, and D. Class D is a five-years' course, at the close of which, on passing a successful examination, the degree of Doctor of Oristry is conferred, which undoubtedly will take high rank as a degree.

Full information respecting the Post-Graduate Dental Association can be obtained by addressing the secretary, Dr. Louis Ottofy, of Chicago, who has given the subject much time and attention. In conclusion, I will state that dentistry is constantly broadening its science and perfecting its art. No limited period of time can be mentioned as sufficient for its acquirement. Like all professions, it will always offer a field for progress and improvement. The establishment of just such post-graduate schools as have been so graphically portrayed to us this evening by the essayist is a "consummation devoutly to be wished." Like the post-graduate medical schools, their tendency would be to elevate the grade of the many practitioners who would be willing to avail themselves of their educational advantages.

*Dr. G. A. Mills.*—Mr. President, as you very kindly invited any one who may be interested in this matter to speak upon it, I will avail myself of the opportunity. About seven weeks ago, Dr. Curtis called my attention to this paper which he intended to read, and asked me if I would be willing to say something upon it, and I told him there was no subject that I would more gladly speak upon.

A man who has been in practice for forty years, as I have been, and who has brought to it a sincere desire to be as good a practitioner as possible, and who in the beginning felt the need of many aids that we now enjoy, will best appreciate the things that come up which promise to be helpful.

This matter of post-graduate schools is a subject that has been very little considered, but which ought to be one of the most important that can come before our profession. It is a subject that was in the mind of one man for a good many years; no one in the profession ever gave to it more thought and attention, probably, than Dr. Atkinson, and he set out to put it in practice in his own way. I think every gentleman here will bear me out in the statement that the matter of clinical instruction which Dr. Atkinson introduced in 1862 was a stepping-stone to this one of post-graduate schools. Every man who has had the advantage of clinical instruction knows how much has been gained by it; yet, while it is a fact that clinical instruction was a great help to us, it falls far short of the demands of to-day. We too well know that clinical instruction is beginning to go out of fashion; it no longer holds the place it did in New York City. I have been in the active movement of the profession, and studied it, for the last thirty years, and I know someting about what it has done in the development of our practice in that time. I accept, in general, the remark that Dr. Curtis puts forth, although he quotes it from another party: that there is nothing truer in relation to the subject of dental education to-day than the fact that the dental profession is largely in a condition of what is called "partial culture." No man will accord greater credit to the schools of our profession than myself. I honor very much the men who have been engaged in them. I think no class have shown a more self-sacrificing spirit than those at work in them; they are dealing earnestly and constantly with the question of dental education, but the very fact that they are so engaged makes it impossible to give the fuller need to the student.

Every man who is conversant with the subject must admit that the average students who go through a course of study in the dental colleges come out lacking in thorough practical instruction. Old Dr. Riggs told a story about a visit of his to Philadelphia, where some boys began asking him questions. One of the young men had a head of large dimensions; the doctor saw what he was aiming at, and he said, "Boys, I have a farm near Hartford, and I have studied on it, among other things, the insect family, and I have noticed the fact that the humble-bee is the biggest when it is hatched." Many students might profit by remembering this story.

There are some questions confronting the dental profession to-day of very grave importance, and they will require wise heads to answer them. Among them is the question of post-graduate instruction and the question of legislation for the benefit of the profession. I have for a long time been an advocate of post-graduate instruction, and I believe it should be brought about in our profession at an early day. We have a great many men who can do some things better than they can do other things, and better, perhaps, than others can do them, and to my mind post-graduate schools would be a good field for that class of men. If a man can do some one thing better than others can do it, let him exercise his talent in that direction. It is a fact that few men are fitted to become general practitioners. If a man takes up one branch of the profession in which he happens to excel he will accomplish more good for the profession and for the public than if he were to give his attention to branches in which, perhaps, other practitioners may excel him. The question of dividing up the profession into specialties has been largely discussed, and there are different opinions upon it; but I think the time is coming when special practice will be more in vogue than it is to-day. We have evidences of it everywhere. We see some men who excel in certain things, and who are more and more devoting their attention to those; one man excels in crown- and bridge-work, another in filling, another in correcting oral deformities, another in oral surgery, and others are more successful in what is called prosthetic dentistry, and their practice falls more and more into those special lines.

Dr. Roosa, of New York, whom I have known for many years, has devoted his attention to developing a post-graduate school in connection with the medical fraternity, and he has brought it to a position of prominence and success. Any man who wants to know anything about the working of post-graduate schools in connection with the medical profession will be able to find it out by examining the methods and results of that school. Dr. Roosa had many difficulties to overcome, but he has energy and ability, and the effort is recognized all over the world as an educational success, while financially the schools are above par. They have the support of the Vanderbilts and other men of wealth, and do not want money for developing hospital helps of their own. A school of that kind in our profession is needed in New York, and if there are men who have the ability and the personality to take hold of such an institution, I have no doubt that there will be found a patronage for it that will carry it to eminent success. I do not know of any place where such a school could be better established than New York City. The time will come when they will be found in many large cities. Dr. Curtis is of the opinion that they would be of very great advantage to the regular schools.

As to Dr. Curtis's idea in regard to making attendance at post-graduate schools obligatory for graduation, I have not considered that point; but I do believe there is an immense advantage to be gained if young men could be brought under such an influence. A school of that kind, to be of any use at all to students and the profession, must be conducted by men who have proved themselves to possess superior ability, and who have had large experience in the practice of their profession,—men who can command respect,—and such men only could expect to receive the patronage of dentists who want something beyond what they have already attained.

The times have changed very much from what they were in former years. The time has been when the student went into the office of a practising dentist, and there had the opportunity to observe operations, and the advantage of the practical instruction of his tutor; but that method of dental education is going out; the schools have instituted new arrangements, taking the student *de novo*, and undertaking to educate him entirely in the college. The result is that the student who comes out of these schools knows very little about his profession in a practical way. There are some young men who will take advantage of every opportunity to push themselves, and who are exceptions to the rule; but the majority of the pupils sent out from the dental schools are unfit to practise. They have to learn by practising on the people who employ and pay them. And it is a fact that the public is beginning to know something about dentistry, and is beginning to discriminate between the men who know and the men who do not know. There is a feeling growing up in the community that the people are being preyed upon too much by young practitioners. I do not say that with any feeling of unfairness towards the young men; it is true that they have to make their way, and they generally do the best they can; but the fact remains that the people do know something about dentists and dentistry, and they prefer the experienced practitioner, if they have to pay for services; therefore young men must embrace every opportunity to prepare themselves for successful practice, and I think that in the establishment of post-graduate schools an opportunity will be given for gaining, in a shorter time, the knowledge and experience that they cannot afford to miss.

*Dr. Luckey.*—Mr. President, I have not very much to say, and there is not a great deal to be said after what we have heard on the subject; but in my humble opinion the place and the time for the work of post-graduate schools is in the primary course of the college. If the colleges already established, and which draw upon the community for the material from which to carve and hew out a supply of dentists for the country, would do their duty by those students that they promise in their catalogues to do, and that the public and the profession expect them to do, there would be no occasion for post-graduate schools, except for the preparation of a few men for some particular lines of work. Post-graduate study we expect every man to follow, but it does not necessarily imply attendance at post-graduate schools. If a member of the profession is found to have a bent for a particular line of work, and feels that he is lacking in thorough equipment, it would be well for him to take a post-graduate course.

I do not think it is necessary to dwell upon this subject. I think the whole thing has been settled in the question of thoroughness in the primary education of the schools.

